# Socio-demographic determinants of low birth weight: Evidence from the Kassena-Nankana districts of the Upper East Region of Ghana

**DOI:** 10.1371/journal.pone.0206207

**Published:** 2018-11-14

**Authors:** Isaiah Awintuen Agorinya, Edmund Wedam Kanmiki, Engelbert Adamwaba Nonterah, Fabrizio Tediosi, James Akazili, Paul Welaga, Daniel Azongo, Abraham Rexford Oduro

**Affiliations:** 1 Navrongo Health Research Centre, Navrongo, Ghana; 2 INDEPTH-Network Secretariat, Accra, Ghana; 3 Swiss Tropical and Public Health Institute, University of Basel, Basel, Switzerland; 4 Regional Institute for Population Studies, University of Ghana, Accra, Ghana; 5 Julius Centre for Health Sciences and Primary Care, UMCU, Utrecht, The Netherlands; University of Zurich, SWITZERLAND

## Abstract

**Objective:**

To examine the social, economic and demographic factors that determine low birth weight in the two Kassena Nankana districts of the Upper East region of Ghana.

**Methods:**

Cross-sectional data was collected from January 2009 to December 2011 using the Navrongo Health and Demographic Surveillance System which monitors routine health and demographic outcomes in the study area. Data on foetal characteristics such as birth weight, and sex and maternal age, parity, maternal education, marital status, ethnicity, religious affiliation and socio-economic characteristics were collected and described. Tests of means, proportions and Chi-squares are employed in bivariate analysis, and adjusted logistic regression models fitted to control for potential confounding variables. All tests were two-sided and test of significance was set at *p-*value of < 0.05.

**Results:**

There were 8,263 live births (44.9% females) with an overall average birth weight of 2.85 kg (2.9 kg for males and 2.8 kg for females). The average maternal age was 28 years, median parity 2, maternal literacy rate was about 70% and 83% of mothers were married. The prevalence of low birth weight was 13.8% 95%CI [13.10, 14.6] and more in female babies than in males (15.5% vs 12.2%; p<0.0001). Determinants of low birth-weight after controlling for confounding factors were sex of neonate (OR = 1.32, 95%CI [1.14,1.52]; p<0.0001), maternal age (p = 0.004), and mothers who are not married (OR = 1.44 [1.19, 1.74]; p<0.0001).

**Conclusion:**

Female neonates in this population were likely to present with low birth weight and maternal factors such as younger age, lower socio-economic status and single parenthood were major determinants of low birth weight. Effective and adequate antenatal care should therefore target women with these risk factors.

## Background

Most low and middle-income countries could not meet the target for Millennium Development Goal 4 (MDG) which is reduction in childhood mortality by two-thirds between 1990 and 2015 [1]. This was in spite of global political, financial and social commitment to the attainment of the MDGs [2]. This calls for more efforts especially in developing countries to unearth context-specific factors that play critical roles in child survival and development.

Birth weight is known to influence the survival and development of neonates. It refers to the first weight measurement taken of a new-born immediately after birth. Normal birth weight is essential for child survival, development and health later in life. According to the World Health Organization (WHO), Low Birth-Weight (LBW) is weight at birth that is less than 2.5 kg [2]. A LBW infant can be born too small (small for gestational age), too early (preterm) or both. Several epidemiological studies have shown that neonates with LBW have a disproportionately higher mortality compared to those with weight higher than 2.5 kg [3]. Even though recent evidences point to declining mortalities among LBW infants, adverse developmental outcomes have still been observed in surviving children [4–6].

A number of studies have reported an association between LBW and pulmonary dysfunction, impaired physical growth, adverse neurological outcome, psycho-social development and social disadvantages [6–9]. An association between LBW and increased respiratory, cognitive, neurological and psychological deficits or dysfunction have also been reported by Kelly et al and Gissler et al [4,8]. Other scholars have observed low school performance, delayed psychomotor development, adverse emotional well-being, as well as increased conduct disorders among children and adolescents with prior LBW [9]. Evidence from life course epidemiological studies indicates that LBW is a major contributor to later development of cardiometabolic diseases such as obesity and atherosclerosis [10]. Further to this, it has been identified as an important contributor to infant and childhood mortality and morbidity [11].

Several studies have reported on the determinants of LBW but these have often not focussed on socio-demographic factors [4]. Non-seasonal factors such as increasing maternal age, socio-economic factors, racial and ethnic differences and availability of health care services have been associated with LBW by several researchers [5,12,13].

Interventions to reduce LBW will require an understanding of the associated foetal, maternal, prevailing environmental and socio-demographic factors. There is however, limited information on this from Northern Ghana. This study examined biological and environmental factors that influence low birth weight in the coverage area of the Navrongo Health and Demographic Surveillance Site (NHDSS) in Upper East region of Ghana.

## Methods

### Study setting

The study was conducted in the Navrongo Health and Demographic Surveillance site. The area lies between latitude 10.300 and 11.100 North and longitude 1.10 West [14]. This area is predominantly Guinea Savannah vegetation with two seasons in a year. It has a short rainy season from April to September and a prolonged dry season from October to March with temperatures ranging from 22.88C to 34.48C [15]. The Navrongo Health and Demographic Surveillance System (NHDSS) is the routine Data collection arm of the Navrongo Health Research Centre which has been in operation in the last 20 years. Ethical clearance was obtained from the Navrongo Health Research Centre Institutional Review Board (NHRC IRB) for the operations of the NHDSS and signed informed consent obtained from all participating households within the study area before data collection. The surveillance area is divided into 5 zone within which are 247 clusters and currently monitors 160, 000 people living in 18,000 compounds (an average of 70 per clusters) and with 32,000 households [14]. The area has one referral district hospital, seven health centres and several primary health-care clinics and 37 Community based Health and Planning Services (CHPS) compounds with resident community health officers providing door-to-door health services to the community.

The study area has a crude birth rate of 25/1000, crude death rate of 10/1000, total fertility rate of 3.8, neonatal mortality rate of 13.4 per 1000 live births, and infant mortality rate of 32.1. Under-five mortality rate per 1000 live births is report as 60.8; life expectancy at birth is 56.4 years for males and 67 years for females [14].

### Study population and sample

The study involved all mothers and their babies born at term (37 weeks and above) from January 2009 to December 2011 and whose parents were resident or natives of the study area. The data was collected through the NHDSS which collects and updates health and socio-demographic characteristics of all persons and households in the two Kassena-Nankana districts of the study area. This involves the regular visits to households by trained fieldworkers every 4 months to interview heads of households or an adult member of the household. Routinely collected data included pregnancies, live and stillbirths, morbidity, deaths, in-migration and out-migrations, childhood vaccinations and verbal autopsy (VAs) on all reported deaths within its operational area. In addition, information on household socio-economic characteristics is also collected.

### Outcome and exposure variables

The main outcome variable for this study is birth weight which was recoded into two mutually exclusive binary outcome: low birth weight defined as birth weight < 2.5 kg and normal birth defined as birth weight ≥ 2.5 kg according to WHO recommendations [16]. Covariates was extracted from the NHDSS data and they included; maternal characteristics such as age, religion, educational status, marital status, parity, and ethnicity. New-born characteristics included date of birth, birth weight and sex at birth. These variables were selected based on the available literature and the potential to influence birth weight. Socio-economic status which is estimated from the wealth index of the household (used as a proxy for household income) was constructed in quintiles (1 = poorest, 2 = poor, 3 = average, 4 = rich, 5 = richest) using Principal Component Analysis (PCA).

Maternal ages at delivery were calculated using the mothers’ and babies’ birthdates. Educational status was defined as either not having any form of formal education or the highest level of formal education attained. Parity refers to the number of births by a mother including the index baby and ethnicity referred to self-reported ethno-linguistic grouping the mother identifies with.

### Data management and analysis

All data are routinely collected using compound registration system, checked for inconsistencies and corrected before they are entered into a Foxpro database. Five per cent of all household data are randomly selected and re-entered as a quality control measure.

The variables were extracted and analysed using the statistical software STATA version 12.0 SE (Stata corp Texas, USA).

Descriptive statistics was used to describe the baseline maternal and new born characteristics. Categorical data are presented in proportions and the differences in socio-demographic characteristics by birth weight status examined using Pearson's Chi squared test.

Determinants of LBW were examined using logistic regression analysis. Reference categories were defined as those usually associated with the lowest birth weight. Odds ratios with 95% confidence intervals are presented and factors associate with LBW are those with a p<0.05. For categorical variables with more than two categories, a post-estimation analysis was done to examine the overall significance of the variable and one p-value obtained. The variance inflation test was employed to test for multi-collinearity between related variables and variables that were found to be collinear were those with a VIF of more than 10 and these excluded from the final logistic regression model.

## Results

Presented in Table 1 are the basic characteristics of neonates and mothers. A total of 8,263 women with singleton babies at term were analysed in this study. More than half (53.3%) of the women were from the Kassena ethnic group. There was a near equal number of female (49.9%) and male (50.1%) neonates. The total average birth weight was 2.85 ±0.52 kg; 2.90 kg for males and 2.80 kg for females. The proportion of LBW was 13.8% 95%CI (13.1, 14.6) with a mean LBW of 1.94 kg (±0.51SD). The proportion of female neonates with LBW was 15.5% 95%CI with an average LBW of 1.95kg (±0.34 SD) and the proportion of LBW among the male neonates was 12.2% (9.4, 15.3) with an overall mean LBW of 1.94 kg (± 0.51SD).

**Table 1:** Descriptive statistics of maternal and neonatal characteristics.

Socio-demographic parameters	LBW (<2500g) (%)	Total	P-value
**Sex of neonate**			
Male	502 (12.2)	4133	<0.0001
Female	641 (15.5)	4130	
Total	1143 (13.8)	8263	
**Maternal age (yrs)**			
15–19	186 (19.8)	939	<0.0001
20–34	759 (13.5)	5628	
35+	198 (11.7)	1696	
Total	1143 (13.8)	8263	
**Maternal parity**			<0.0001
1	498 (20.5)	2429	
2–3	360 (11.0)	3265	
4+	285 (11.1)	2569	
Total	1143 (13.8)	8263	
**Maternal Education**			
No formal education	293 (12.8)	2293	0.080
Primary	311 (12.5)	2483	
JHS	265 (15.1)	1761	
SHS	105 (14.7)	716	
Tertiary	41 (15.4)	266	
**Total	1015 (13.5)	7519	
**Socio-economic status**			
Poor	218 (12.2)	1794	0.015
Next poor	214 (14.7)	1454	
Average	222 (16.1)	1383	
Next rich	232 (13.7)	1698	
Rich	180 (12.8)	1408	
Missing	77 (14.6)	526	
**Marital Status**			<0.0001
Married	711 (12.2)	5845	
Not Married	224 (19.2)	1170	
**Total	935 (13.3)	7015	
**Ethnicity** Kasena	590 (14.3)	4118(100)	0.197
Kasem	590 (14.3)	4118	
Nankana	412 (12.9)	3196	
Builsa	34 (16.8)	203	
Others	30 (13.6)	221	
**Total	1066 (13.8)	7738	
**Religion**			
Traditional	395 (13.7)	2885	
Catholic	271 (14.3)	1898	
Other Christians	304 (13.3)	2282	0.833
Islam	94 14.4)	651	
Other	2 (9.1)	22	
**Total	1066 (13.8)	7738	

** Total not 8263 due to missing values in the variables

The average maternal age was 28 years (±7.4 SD), about 11% of the mothers were teenagers, about 31% had not attained any formal education, about 23% of the mothers were in the poorest quintile and 18% in the rich quintile. 83% of the mothers were married.

A large fraction of the LBW outcomes 19.8% (17.3, 22.5) were from mothers in the age group 15–19 years and the least from mothers aged 35 plus 11.7% (10.2, 13.3). Large proportion (20.5%) of mothers, who previously had one child, had higher LBW children compared to those who had between 2–3 children (20.5% vs 11.0%). The proportion was still high when compared with their counterparts who previously had 4 or more children (20.5% vs 11.1%). Mothers who belong to a household in the upper socio-economic class (Rich, 12.8% & Richest, 13.7%) and lower socio-economic (Poor, 12.2% & Poorest, 14.7%) class had comparable proportion of birth weights, but those who were in the middle class (Average) had large proportion (16%) of low weight babies (Pearson chi2(4) = 12.2861 P-value = 0.015).

### Bivariable and multivariable logistic regression analysis

An initial unadjusted bivariable analysis of the maternal and socio-demographic characteristics showed that sex of neonate, maternal age, household socio-economic status and marital status significantly influenced the birth weight of a neonate as shown in Table 2.

**Table 2:** Maternal, demographic and socio-economic factors associated with LBW in the Kassena-Nankana districts.

Determinants	Unadjusted OR (95% CI)	P-value	Adjusted OR (95% CI)	P-value
**Sex of Child**				
Male	1		1	
Female	1.33 (1.17, 1.51)	<0.000	1.32 (1.14, 1.52)	<0.001
**Mothers age**				
15–19 yrs	1		1	
20-34yrs	0.63 (0.53, 0.75)	<0.000	0.69 (0.55, 0.87)	0.004
35+ yrs	0.54 (0.43, 0.67)		0.65 (0.49, 0.85)	
**Mother education**				
No education	1		1	
Primary	0.98 (0.82, 1.16)	0.001	0.88 (0.73, 1.06)	0.190
JHS	1.21 (1.01, 1.45)		1.08 (0.88, 1.33)	
SHS	1.17 (0.92, 1.49)		1.16 (0.87, 1.54)	
Tertiary	1.24 (0.87, 1.77)		1.49 (0.95, 2.34)	
**Socio-economic status**				
Poorest	1		1	
Poor	0.80 (0.65, 0.98	0.016	0.81 (0.65, 1.02)	0.002
Average	1.11(0.90, 1.36)		1.13 (0.91, 1.42)	
Rich	0.85 (0.69, 1.05)		0.65 (0.49, 0.87)	
Richest	0.92 (0.75, 1.12)		0.86 (0.69, 1.09)	
**Marital status**				
Married	1		1	
Not married	1.71 (1.45, 2.02)	<0.001	1.44 (1.19, 1.74)	<0.01
**Ethnicity**				
Kasenna	1		1	
Nankana	0.88 (0.77, 1.01)	0.198	0.86 (0.73, 1.01)	0.139
Builsa	1.20 (0.82, 1.76)		1.19 (0.79, 1.82)	
Others	0.94 (0.63, 1.39)		0.21 (0.41, 1.30)	
**Religion**				
Traditional	1		1	
Catholic	1.05 (0.89, 1.24)	0.843	0.99 (0.82, 1.22)	0.711
Other Christian	0.97 (0.82, 1.14)		0.93 (0.77, 1.13)	
Islam	1.06 (0.83, 1.36)		1.11 (0.81, 1.53)	
Others	0.63 (0.15, 2.71)		1.05 (0.24, 4.69)	

Multicollinearity was detected between maternal age and parity after performing a variance inflation test (VIF) and thus parity was not included in the adjusted logistic regression model.

Factors associated with LBW from the multivariable analyses included sex of neonate, maternal age, and marital status. Female neonates had about 32% increased odds of being born with low birth weights compared to their male colleagues; (OR = 1.32 95%CI [1.14, 1.52]; p < 0.0001).

Mothers in the 20-34-year age group were less likely to have neonates with LBW compared to those below 20 years (OR = 0.69 95% CI 0.55, 0.87, p = 0.004). Similarly, the odd of having LBW neonate was 35% (OR = 0.65, 95% CI 0.49, 0.85, p-value = 0.002) lower among women who were 35 years and above when compared to the under 20 years group. Socio-economic condition as measured in this study was an important risk factor for LBW as identified by other studies in similar settings [17,18]. Our results also suggest that more non-married women are prone to having babies with LBW than married women.

## Discussions

In this study, we have examined how socio-demographic, foetal and maternal factors predict low birth weights of new-born babies in the Kassena-Nankana districts in northern Ghana. We found that the mean birth weight of babies in the study area was 2.85 ±1.0 kg with males significantly heavier than females by 0.1 kg. Our sample size was powered enough to detect small but significant difference in the birth weight of male and female babies. Some studies [17,18] also found similar results where males were found to weigh heavier than boys at birth. Voldner et al. in their study accessing the determinants of birth weight for boys and girls at birth, found boys to be heavier than girls. They explained that, paternal birth weight significantly influenced the weight of boys but not for girls and further suggested that there is a genetic regulation along the male line [19]. We also found out that the prevalence of low birth weight in the study area (13.8%) was higher than the national prevalence of 10.7% [20]. Though the national prevalence was reported as 10.7%, the administrative regional prevalence varied considerably. Both our study and that of Manyeh et al, 2016 had prevalence comparable to the estimates of the administrative regions where the study sites are located. That is, 13.8% in our study site vs 14.5% in the Upper East region and 7.9% in Dodowa HDSS vs 9.9% in the Greater Accra region. However, Abubakari (2015) in their study in Northern region, a similar setting as ours, found prevalence of LBW nearly twice (26% vs 11.9%) when compared to the regional estimate. The large difference observed in Abubakari [17] and the regional estimate compared to our study and Manyeh et al, 2016 could be attributed to methodological differences. Both our study and Manyeh et al, 2016 utilized data from health and demographic surveillance sites where data is collected at the population level whilst Abubakari (2015) relied-on data from some selected health facilities using systematic sampling approach. The northern part of Ghana is classified as the poorest regions [21], while Accra located in the southern part classified as the richest. This might explain why the proportions reported by Manyeh (2016) for the southern part of Ghana are considerable lower than what we found in our study in northern Ghana since socio-economic status influence birth weight.

Determinants of LBW in this population were maternal age, lower socio-economic status and single parenthood. Among these, maternal age constituted an important risk factor with women less than 20 years of age recording the highest proportion (19.8%) of LBW born in this study. The strong association between maternal age and birth weight is consistent with a study conducted by MacLeod and Kielyin 1988 where they found a significant progression of birth weight with advancing age of mother [22]. Similar results were obtained by Dičkute et al, 2004 who in their study in Lithuania identified a U-shaped relationship between maternal age and LBW risk consistent with the under 20 year group of this study but differed in the 35 year group of this study where higher proportions of LBW were recorded for under 20 years and 20–34 years but lowest for the above 35 years group [23,24]. Some studies have argued that the negative effect of a higher maternal age can be weakened by maternal education [23,25] which is likely to be the case with the low proportion of LBW recorded for the 35 year age group for this study.

Both bivariable and multivariable analyses did not show any significant association between birth weight and educational status of the mother, this contrast studies conducted in two different political and social systems, West and East Germany. They found that mothers among the lowest category of education in both West and East Germany had an unadjusted relative risk of 2.5 for delivering an small for gestational age child compared to those of the highest education category [26]. Both the chi square test of association and the multivariable logistic regression model study found a significant association between marital status and birth weight of a neonate. Being married was found to be protective against low birth weight in our findings and those who were not married had 71% increased odds (OR = 1.71) of having a neonate with low birth weight. This is consistent with a study by Foix L'Helias and their work where they found risk factors including being single [27] to low birth weight among neonates. However, a study from the Dodowa HDSS found no relationship between birth weight and marital status, this could be attributed to methodological difference in the classification of the marital status variable (i.e. two variables vs four variables) [18]. Also difference in socio-cultural practices in terms of marriages between the northern and southern parts of Ghana could account for these differences.

Both regression analyses and chi squared test did not show any association between religious affiliation of the mother and birth weight. This is contrary to Burdette *et al*. in their examination of maternal religious attendance and low birth weight in the United States where they identified maternal religious attendance to be protective against low birth weight and stated that lower rates of cigarette use help to mediate 11% of the association between religious affiliation and low birth weight. Their results further suggested that the health benefits of religious involvement have the potency to extend across generations [28]. However, this was not the case in our study, this can be explained by the fact that smoking is not a common practice and in most cases a taboo for women in rural Ghanaian setting. Household socio-economic status was significantly associated with low birth weight at both the bivariable and multivariable analyses. LBW could be due to poverty which is a consequence of poor socio-economic status. Our findings are consistent with some previous studies conducted elsewhere [29,30]. Similar findings were reported in a study from southern rural Ghana [18]. Spencer et al in 1999 concluded in their study that, a substantial proportion of births below 2.5 kg and below 1.5 kg are attributable to social inequality, their results demonstrated the likelihood of being born weighing more than 3.5 kg if one comes from socially advantaged group [30]. Ethnicity was not associated with birth weight. This is not surprising since there is not much difference in the socio-cultural practices among the major ethnic groups in the study area.

Neonatal sex is a very important risk factor identified by this study as it was highly associated in all levels of the analysis. Female neonates had 33% increased odds (OR = 1.33 95%CI [1.17, 1.51]) of being born with LBW than their male counterparts. Manyeh (2016), found that being born female was protective against LBW in southern Ghana while Abubakari (2015) on the contrast found similar results with our study where being a male neonate was rather protective against LBW in rural Northern Ghana [17,18]. The southern and northern parts of Ghana are geographically and ethnically different and this might explain the difference observed. Further studies will be needed in this regard to establish the variation of neonatal sex as a risk factor of LBW in the northern and southern parts of Ghana.

LBW continues to be a significant public health problem and as multiple factors are associated with it, it requires a more holistic and multipronged approach for its reduction. The concept of high-risk approach needs to be implemented which means better health care services to all antenatal subjects with special attention to those who are found to be at high risk. Early registration of pregnancy should be promoted so as to detect the presence of any high-risk factors at the earliest. Importance of regular ANC visits should be explained to each of the high-risk women so that any untoward consequences can be averted. Strengthening Information-Education-Counselling activities at health centres and/or CHPS compounds and in the community would help to a great extent. Such education must address issues like harms of early marriage, teenage pregnancy and proper nutrition during pregnancy.

## Conclusion

Female neonates in this population were likely to present with low birth weight and maternal factors such as younger age, lower socio-economic status and single parenthood were major determinants of low birth weight. Effective and adequate antenatal care should therefore target women with these risk factors.

## Supporting information

S1 Data De-identified dataset. (XLS)

S1 Table Generalized estimating equation for birth weight adjusting for cluster effects. (DOCX)
