# Supplementary material for: Socio-demographic determinants of low birth weight: Evidence from the Kassena-Nankana districts of the Upper East Region of Ghana
Source: PLoS One. 2018 Nov 14;13(11):e0206207. doi: 10.1371/journal.pone.0206207 (PMC6235265; doi:10.1371/journal.pone.0206207)
Supplement: S1 Table — (DOCX) [file pone.0206207.s002.docx]

**Generalized estimating equation for birth weight data adjusting for cluster effects**

| **Determinants** | **Coefficient**  **(95% CI)** | **P-value** |
| --- | --- | --- |
| **Sex of Child** |  |  |
| Male | 1 |  |
| Female | 0.228 (0.111, 0.345) | <0.000 |
| **Mothers age** |  |  |
| 15-19 yrs | 1 |  |
| 20-34yrs | -0.344 (-0.525, -0.162) | <0.000 |
| 35+ yrs | -0.400 (-0.617, 0.199) |  |
| **Mother education** |  |  |
| No education | 1 |  |
| Primary | -0.087 (-0.253, 1-0.079) | 0.303 |
| JHS | 0.027 (-0.146, 1.45) | 0.761 |
| SHS | 0.127 (-0.111, 0.361) | 0.303 |
| Tertiary | 0.309 (-0.093, 0.711) | 0.131 |
| **Socio-economic status** |  |  |
| Poorest | 1 |  |
| Poor | -0.202 (-0.415, 0.011) | 0.063 |
| Average | 0.064 (-0.147, 0.275) | 0.552 |
| Rich | -0.358 (-0.618, -0.097) | 0.007 |
| Richest | -0.127 (-0.318, 0.065) | 0.196 |
| **Marital status** |  |  |
| Married | 1 |  |
| Not married | 0.294 (0.157, 0.430) | <0.001 |
| **Ethnicity** |  |  |
| Kasenna | 1 |  |
| Nankana | -0.118 (-0.252, 0.016) | 0.085 |
| Builsa | 0.211 (-0.176, 0.597) | 0.285 |
| Others | -0.182 (-0.618, 0.255) | 0.415 |
| **Religion** |  |  |
| Traditional | 1 |  |
| Catholic | 0.026 (-0.147, 0.198) | 0.771 |
| Other Christian | -0.067 (-0.223, 0.089) | 0.400 |
| Islam | 0.074 (-0.193, 0.342) | 0.588 |
| Others | -0.158 (-1.328, 1.012) | 0.791 |
